# Infamous Attack of the Nashville Journal upon Dr. S. D. Gross, and the Jefferson Medical College

**Published:** 1858-09

**Authors:** 


### ARTICLE VI.

*Infamous attack of the Editor of the Nashville Journal, upon Dr. S. D. Gross, and the Jefferson Medical College.* By MOXA.

We have been no little surprised and aggrieved at the appearance in the Nashville Journal, of quite an elaborate editoral, devoted to Dr. Gross and the Jefferson Medical College, to which we wish to direct attention.

The article seems to have been called forth, from the appearance in the N. A. Medico-Chirurgical Review, of an editorial in relation to the late meeting of the American Medical Association, in which Dr. Gross has dared to oppose the views of the editor of the Nashville Journal, in his criticism upon the address of the retiring President, Dr. Eve. Truly has this "Yankee Blade" and "Halifax handle," * most horribly pricked the gasometer of this gasconader, as proven by the odorous escape of fifteen pages of filthy abuse.

We beg Dr. Gross to be more careful in piercing his "Yankee Blade" into such gasometers, and filling with a most horrible stench the nostrils of the profession.

* The quotations in this article, are beautiful and elegant extracts from the Nashville Journal.

We are not surprised that this wonderful *hybrid* should become somewhat aroused, when the ridiculous position of his confrere upon the *moral* power of the Association is so plainly shown up—that he should raise a loud *bray*, from the thrust of this keen "blade" of the Review.

In the whole length and breadth of *the fifteen pages*, we find not a single argument to controvert the criticisms of Dr. Gross, but in lieu thereof, the most unjustifiable abuse, in a style truly characteristic of Dr. Bowling, an example of which we give in the following: "Had one of the Egyptian snakes crept out of the anus of the snake of Moses, and, finding itself only half digested, proposed to feed on snakes the balance of its life, his snakeship would have been guilty of folly less sublime in height and depth than that of Dr. Gross." We think Dr. B. has shown an inconsistency unusual with the most of black-guards, in turning his battery upon his superiors; they usually have the redeeming feature of seeking their equals or inferiors. Had Dr. B. made the following comparison, we believe he too would have availed himself of this redeeming quality, the only one we ever knew his class to be tıoubled with.

Who is Dr. Bowling?

The *distinguished* author of the "DANCE OF BITCHES," a most unpardonable gasconader—the would be Medical Regulator—and editor Sen., of the (notoriously vulgar) Nashville Journal.

Who is Dr. Gross?

We do not wonder at the distinguished author calling him a "*Blade*," as he undoubtedly has felt its keen point. Author of Gross's Pathological Anatomy, one of the most popular and able of all American Medical works. A standard authority, sufficient *alone* to entitle him to the front rank in the list of American Medical men. Editor, Sen., of one of the most ably conducted periodicals of this or any other country. A man whose towering genius has given to American Medicine some of its brightest laurels.

"The horizen of the one," (Dr. Eve,) "from the altitude of his stand point must forever remain a region of glory shut out from the vision of the other," (Dr. Gross.)

No man, save him, whose face is brass, and whose brain is gas, but would have known, that such an idea as the one above quoted, would have produced among all sensible Medical men, at least in Georgia, only ridicule and laughter.

We do think that Dr. Bowling sustains the reputation and position he has acquired with more ability than any living man, viz: the GREAT MEDICAL GASCONADER.

"We ask every member of the American Medical Association present at the last meeting, if it is not his deliberate opinion, that had Dr. Gross embodied in a speech before that body the editorial we have quoted, would he not the same hour have been expelled."

Wonderful Association, in our boasted free land, that would expel a member for discussing its powers—*brilliant idea.* It is not strange, however, that the *Gasconader* thinks so, for he unquestionably feels himself and colleague (Dr. Eve,) the embodiment of the power both moral, mental, and legislative of the Medical Association.

The editorial of Dr. Gross, the subject of so much ireful indignation, at the next meeting of the Association, will stand upon record, and we hope *Dr. Bowling* will not fail to present this *grave* crime to the consideration of that august assemblage.

The American Medical Association, is not the representative of the Profession at large, neither is it the representative of the Medical Colleges of the Nation, and consequently has no power over either. It is presumption and folly for any organized body to assume power over that which it does not represent.

"He" (Dr. Gross,) "puts on airs," &c., "and dares even to dictate terms to the American Medical Association."

The *Medical Regulator*, would not quietly submit thus to have the palm of glory torn from his own brow, else would he be a most "egregious madman." Every reader of the Nashville Journal, knows full well that its editor, has long since, assumed to himself the dictatorship, not only of the American Medical Association, but of the Profession at large,

"He" (Dr. Gross,) "takes to himself the credit of multiplying Schools in the South, in order to break up established Institutions, that the Jefferson Medical College, may become the tictac for the multitude of students of the South to graduate or *buy a Diploma at.*" *

We pronounce this an intentional and infamous libel upon Dr. Gross and the Institution with which he is connected.

It was our good fortune in times gone, to have been a student of the Jefferson Medical College, and we speak advisedly.

There are some, among the thousands who boast of having been educated in her halls, who will not supinely be, while her fair name is being defamed by this *braying hybrid*, of the Nashville Journal. Her many bright laurels are still resting upon her brow, and time itself will not wear them away, and the traitor who by malice and jealousy attempts to pluck from her wreath a single flower, should receive the treatment due a traitor's conduct. Dr. Bowling was, we believe, nurtured under the wing of the man whom he maligns, and now Arnold-like, he turns his battery upon his old preceptor. "Oh, shame where is thy blush!" It is true Dr. Gross is a "new cog," and the workmen who placed him in the *mighty wheel*, understand well their business. This "new cog," only gives still brighter lustre to the material which passes through the Jefferson Medical College.

"*Or buy a Diploma at.*"

What "insufferable arrogance." What an "egregious madman." What a wholesale slanderer. Had this false insinuation any foundation in truth, it would not compare in contemptible littleness with *the peddling of Medical Tickets over the country with a view of swelling the number of a class.*

"The insolence of the Review, in declaring that the Medical power is with the schools, &c."

The insolent arrogance of this distinguished author and colossal Gasconader, wholly disables him to appreciate the opinions of any save himself.

---

* Italics ours.

"O wad some pow'r the giftie gie us
To see oursels as others see us!
It wad frae monie a blunder free us,
And foolish notion."

As before remarked, we contend that the National Association has *no power*, either moral or otherwise, from the fact that it is not the representative of the colleges or the profession. It may elect officers, pass resolutions, appoint time and place of annual meetings, make recommendations, enjoy festivities, &c. This covers the breadth and depth of its power, even with such a colossus at its helm as Dr. Bowling, who, by the way, should be its next president, as he considers it a most undying honor, and so it would be to him—upon Dr. Gross, it would be none, neither would the Presidency have been to Clay, Calhoun or Webster. Dr. Gross would *honor* the association as its President, but the position would not honor him. Dr. B. endeavors to flatter Southern pride by calling Dr. Gross a "northern man." True, and were he invested with the most inveterate abolition principles, he would be a purer man than Dr. Bowling. The Southern patriot will not be so easily gulled, by this base insinuation contained in the reference to Dr. Gross as a "northern man." He is of the North and one of her brightest luminaries, more than can be said of *our* distinguished author of the "DANCE OF BITCHES," in connection with any locality. Our own Carolina Dickson, Georgia Meigs, with the *adopted* Dunglison, and the "northern Gross," Pancoast and others, form a combination of intellect and power unexcelled, the *truth and force* of which the editor of the Nashville Journal may one day feel. Would Dr. B. do us the favor to send a copy of one of the "Text books," of the Professor of Practice, in the Nashville School, ("*Dance of Bitches*,") to Robley Dunglison for his edification and enlightenment. "If the lay members of the South" and North "do not lay" you Dr. B. "where you deserve for your arrogance, to be laid, we will acknowledge our ignorance of their metal." We think, however, from the stride recently taken by the Dr. that he will readily seek that position without assistance; though his lately plucked feathers have grown quite rapidly to be thus early spreading to the breeze, for his own admiring gaze, yet he should be careful they do not fall (as in by-gone times) upon being shown his ugly features, as instantly as the gaudy plume of the Pea Cock upon beholding the ungraceful appearance of his pedestrial extremity.

"*Dr. Gross jealous of Eve*;" was the Bull jealous of the gnat which sat upon his horn and with swelled importance intimated that he would leave if his weight were troublesome.

"Dr. Gross owed his place upon his return from New York to Louisville, to the *noble*, *self-sacrificing* generosity of Eve." Very self-sacrificing generosity to give up a place the term of which had expired, with the purpose of regaining his lost Professorship in Augusta, failing in which, he became the enemy of Dr. Dugas, who had been elected to fill the vacant chair, and the Georgia Medical College, because he could not *force* a vacancy.

"Again—It has been insinuated that he did not join the Faculty here, (Nashville,) the year after he gave his chair at Louisville to Dr. Gross, until a large bonus had been assured him."

"*Gave his Chair at Louisville to Dr. Gross.*" If we are not mistaken, the Trustees of that Institution elected Dr. Gross to fill the *vacancy*, which occurred by the close of the term for which Dr. Eve was *employed.* Upon the most reliable authority, we state our belief that *Dr. Eve went to Nashville upon a bonus* the first session, and we have reason to believe that he remained the next year upon the same conditions.

From certain indications there are good reasons for saying —"It is prophecy now," that the *self-sacrificing* Eve of the Nashville School, will look for better quarters, since this boasted Institution is so rapidly on the decline. The Nashville College has been afflicted with such an immense gaseous inflation, we are not, however, astonished at its loss of dimensions. "But at last Dr. Gross is a President," of a Society, composed no doubt of men of science and ability—

a position perhaps as honorable as that of the author of a certain, *Canine work.*

Dr. Gross is promised another hearing from the immortal "D. of B." when the weather gets cooler. In our opinion the renowned author, will still be feeling hot when the winds of winter are whistling about him, for the few sinapisms we are now applying, are only indications of the general vesicating process his carcass may undergo. It is prophecy now, but ere the first of another year it *will be history.*

More than two years since we saw the Nashville School so swelled with arrogant presumption and inflated bombast, we predicted its decline. *It is now history.* For the session of 1856 and 57, the Nashville School numbered 419 matriculants including 40 M. D's. The session of 1857 and 58, the class numbered 353 matriculants including 41 M. D's., making a decrease of 66 students, even with the assistance of an increased number of matriculating M. D's. "This is history."

In three more sessions, making the ten years which the prophet and author has given the Nashville School to become the "great centre of Medical teaching," then will be *the history* which has long since been predicted, that "Pride cometh before a *fall.*"

We hope we shall hear more from this *vaunting bombast*, this *braying hybrid*, this CANINE, B; we have a plaster of larger dimensions and greater vesicating power that we are anxious to apply "when the weather gets a little cooler."

In conclusion, we would remark, that this style of writing is not to our taste and not the best suited to the pages of a Medical Journal, but is adopted as the only one suited to this miserable compound of intrigue, selfishness and billingsgate embodied in the individual who has made this most uncalled for and unjustifiable assault upon one of the most eminent Medical men of the United States and upon one of the oldest and most prominent Medical Institutions of the country.

For reasons, which are satisfactory to the writer, the name of the author is not attached to this communication, but is left with the editor and can be readily obtained upon the proper application.

P. S. We would suggest the idea of a rotten bottom in the "tic-tac tub mill" at Nashville, (so called *University*, save the mark;) which might well be replaced by an entirely new machine.

